# Complete Genome Analysis Reveals the Quorum Sensing-Related Spoilage Potential of *Pseudomonas fluorescens* PF08, a Specific Spoilage Organism of Turbot (*Scophthalmus maximus*)

**DOI:** 10.3389/fmicb.2022.856802

**Published:** 2022-04-18

**Authors:** Dangfeng Wang, Fangchao Cui, Likun Ren, Xiqian Tan, Xinran Lv, Qiuying Li, Jianrong Li, Tingting Li

**Affiliations:** ^1^School of Food Science and Technology, Jiangnan University, Wuxi, China; ^2^College of Food Science and Technology, Bohai University, Jinzhou, China; ^3^National & Local Joint Engineering Research Center of Storage, Processing and Safety Control Technology for Fresh Agricultural and Aquatic Products, Jinzhou, China; ^4^Key Laboratory of Food Science and Engineering of Heilongjiang Province, College of Food Engineering, Harbin University of Commerce, Harbin, China; ^5^Key Laboratory of Biotechnology and Bioresources Utilization, Ministry of Education, Dalian Minzu University, Dalian, China

**Keywords:** *Pseudomonas fluorescens* PF08, complete genome, spoilage ability, quorum sensing, specific spoilage organism

## Abstract

*Pseudomonas fluorescens* is a common specific spoilage organism (SSO) of aquatic products. The spoilage ability of SSO can be regulated by the quorum sensing (QS) system. However, the QS system in *P. fluorescens* and their relationship with the spoilage potential have not been systematically analyzed. In the present study, the complete genome of *P. fluorescens* PF08 isolated from spoilage turbot was sequenced. The identification of key genes that involved in the QS, enzyme synthesis, sulfur, and amino acid metabolism explained the spoilage potential of *P. fluorescens* PF08. Results of quantitative real-time PCR revealed the key role of the *P. fluorescens* PF08 QS system in regulating the transcription of spoilage-related genes and its sensitivity to environmental stress. These findings provide insight into the spoilage features of *P. fluorescens* PF08 from a genomic perspective. The knowledge may be valuable in the development of new strategies for the targeted inhibition of aquatic product spoilage based on QS interference.

## Introduction

Food spoilage is a complex process that is caused mainly by the metabolic activities of specific spoilage organisms (SSOs). SSOs are regulated by a common cell-to-cell communication mechanism named quorum sensing (QS) (Bai and Rai, 2011). In the food industry, microbial spoilage has attracted increasing attention because of the subsequent economic losses and safety problems. When the self-secreted QS signaling molecules reach a certain threshold level with the increased density of the contaminating bacteria during food storage, bacteria will regulate the secretion of a series of spoilage-required factors such as extracellular proteases and lipases, resulting in protein degradation and off-flavor in food (Papenfort and Bassler, 2016). Detection of the key regulators of the QS system in spoilage bacteria and blocking or interfering with the operation of the system is a feasible strategy to control food spoilage while avoiding drug resistance caused by the survival pressure (Ding et al., 2017).

*Pseudomonas fluorescens* is a common Gram-negative bacterium and a major spoilage organism frequently associated with the spoilage of aquatic products (Speranza et al., 2013). Moreover, *P. fluorescens* is a psychrotroph and can grow well at temperatures as low as 4°C. Thus, spoilage caused by *P. fluorescens* cannot be effectively controlled by the low temperature environment in cold chain transportation (Packiavathy et al., 2014). Similar to most Gram-negative bacteria, the secretion of spoilage factors in *P. fluorescens* can be regulated by QS based on *N*-acyl-homoserine lactones (AHLs) (Brachmann et al., 2013). The genetic background and genomic information of the QS system have been intensively studied in *P. aeruginosa* (Chan et al., 2014), but little related information is available for *P. fluorescens*. The availability of the genome sequence of *P. fluorescens* would be helpful to clarify the key factors of QS-related system in *P. fluorescens*. This information could also be expected to improve the understanding of the QS-regulated spoilage in *P. fluorescens* and to inform new strategies for quality control of aquatic products.

The genomes of different *P. fluorescens* strains have been sequenced and uploaded to the Genome database of GeneBank^1^. Most of the genomic information came from *P. fluorescens* isolated from soil, sewage, or the plant rhizosphere. There is scant information concerning the relationship between the genome information and food spoilage. Therefore, in the present study, the entire genome of *P. fluorescens* strain PF08, which we previously reported as the SSO of refrigerated turbot (*Scophthalmus maximus*) (Li T. et al., 2019), was sequenced and analyzed. Several QS-related genes were found in the *P. fluorescens* PF08 genome. Analysis of the genome identified several genes and gene clusters involved in the protease production, sulfur, and amino acid metabolism, stress response, biofilm formation, motility, and drug resistance. The findings revealed the spoilage potential of PF08 and its excellent adaptability to external survival pressure exerted by food preservation. Moreover, the QS system in PF08 was inhibited or activated by cinnamaldehyde or C_4_-HSL in this study and the regulatory effect of the QS system on spoilage-related genes in PF08 was evaluated by analyzing the changes of transcription level of these key genes. The effects of environmental conditions on the transcription of QS regulatory genes of *rhlI/R* were also determined. The data provide insight into the spoilage features of *P. fluorescens* PF08 from a genomic perspective.

## Materials and Methods

### Materials and Bacterial Strains

The *N*-butanoyl-L-homoserine lactone (C_4_-HSL) standard was purchased from Sigma-Aldrich (St. Louis, MO, United States). It was dissolved in methanol at a concentration of 2 mg/mL for use.

*Pseudomonas fluorescens* PF08 (GenBank accession number: CP032618) was isolated from refrigerated spoiled turbots in Jinzhou, China. *Aeromonas salmonicida* CS12 and *Staphylococcus aureus* were kept in our laboratory. All the bacteria were cultured in Luria-Bertani (LB) broth (1% peptone, 1% NaCl, 0.5% yeast extract, pH 7.0 ± 0.2) at 28°C with agitation (160 rpm) until an optical density at 595 nm (OD_595_) of 1 was reached.

### Genome Sequencing and Assembly

Ten mL of overnight culture of *P. fluorescens* PF08 (OD_595_ = 1) was centrifugated at 8000 × *g* for 10 min. Then, the cells were collected and transported to the Beijing Novogene Bioinformatics Technology Co., Ltd., in dry ice for genome sequencing and assembly. The experimental details are described in the Supporting Information.

### Genome Component Prediction and Functional Annotation

The interspersed repetitive sequences of the genome were predicted using RepeatMasker (Saha et al., 2008)^2^. Tandem repeats were analyzed using the tandem repeats finder. Transfer RNA (tRNA) genes were predicted using the tRNAscan-SE (Lowe and Eddy, 1997). Ribosomal RNA (rRNA) genes were analyzed using the rRNAmmer (Lagesen et al., 2007). The IslandPath-DIOMB program (Hsiao et al., 2003) was used to predict genomics islands. Transposon PSI was used to predict transposons based on the homologous Blast method. CRISPRFinder was used for the CRISPR identification (Grissa et al., 2007).

The gene functions of the genome were annotated through Blast searching the whole genome against seven databases (*E*-value < 1e-5, minimal 30 alignment length percentage > 40%), including the Gene Ontology (GO), Kyoto Encyclopedia of Genes and Genomes (KEGG), Clusters of Orthologous Groups (COG), Non-Redundant (NR) protein databases, Transporter Classification Database (TCDB), Swiss-Prot, and TrEMBL. The Antibiotic Resistance Genes Database (ARDB) was used for drug resistance analysis.

### Phylogenetic Analysis

A multiple alignment of whole genome sequences from PF08 and other *Pseudomonas* strains compared in this analysis was generated using CVTree 3. The tree was constructed using the alignment-free method based on K-tuple counting and background subtraction with a bootstrap value of 1,000.

### Effects of Quorum Sensing System on the Transcription of Spoilage-Related Genes in PF08

An overnight culture of *P. fluorescens* PF08 (OD_595_ = 1) was diluted 1:100 in fresh LB broth (pH 7.0 ± 0.2). C_4_-HSL, and cinnamaldehyde were used as the agonist and inhibitor of the QS system and added to the broth at final concentration of 20 μg/mL or 0.1 μL/mL, respectively (Li et al., 2018). The broth containing both of 20 μg/mL of C_4_-HSL and 0.1 μL/mL of cinnamaldehyde was used as control group. The samples were incubated at 28°C until OD_595_ was 1. After centrifugation at 8000 × *g* for 10 min, the pelleted cells were examined to determine the transcription levels of key genes (*rhlI*, *rhlR*, *pilG*, *pilJ*, *fliL*, *flhA*, *algA*, *algX*, *aprX*, *aprA*, *serP*, *pvdA*, *pvdE*, *ahpC*, *sspA*, *cusS*) using quantitative real-time (qPCR). Samples incubated in LB broth containing equivalent concentration of methanol at 28°C were the blank group.

### Effects of Environmental Conditions on the Transcription of *rhlI/R* System in PF08

#### Carbon Sources

AB medium (3 g/L K_2_HPO_4_, 0.3 g/L MgSO_4_⋅7H_2_O, 1 g/L NH_4_Cl, 0.15 g/L KCl, 1 g/L NaH_2_PO_4_, 0.01 g/L CaCl_2_, 0.0025 g/L FeSO_4_⋅7H_2_O, 5 g/L casein hydrolyzed amino acids) was prepared as previously described (Zimmer et al., 2014). The overnight culture of *P. fluorescens* PF08 (OD_595_ = 1) was diluted 1:100 in prepared AB culture medium, followed by the addition of 5 g/L of different carbon sources (glucose, xylose, sucrose, fructose, lactose, or maltose). All samples were incubated at 28°C until an OD_595_ of 1 was reached. After centrifugation at 8000 × *g* for 10 min, the cells were collected for the analysis of *rhlI/R* transcription level using qPCR. Samples incubated in LB broth at 28°C were the control.

#### NaCl

An overnight culture of *P. fluorescens* PF08 (OD_595_ = 1) was diluted 1:100 in fresh LB broth (pH 7.0 ± 0.2) containing different mass concentrations of NaCl (0.5, 1, 3, or 5%). All samples were incubated at 28°C until an OD_595_ of 1 was reached. After centrifugation at 8000 × *g* for 10 min, the precipitates were collected for further analysis of *rhlI/R* transcription level using qPCR.

#### pH

An overnight culture of *P. fluorescens* PF08 (OD_595_ = 1) was diluted 1:100 in fresh LB broth. The pH was adjusted to 5, 6, 7, 8, or 9 by addition of HCl or NaOH. All samples were incubated at 28°C until OD_595_ was 1. After centrifugation at 8,000 × *g* for 10 min, the cells were collected for further analysis of *rhlI/R* transcription level using qPCR.

#### Temperature

An overnight culture of *P. fluorescens* PF08 (OD_595_ = 1) was diluted 1:100 in fresh LB broth (pH 7.0 ± 0.2). The samples were incubated at 4, 15, 25, or 37°C until OD_595_ was 1. After centrifugation at 8000 × *g* for 10 min, the cells were collected for further analysis of *rhlI/R* transcription level using qPCR. Samples incubated in LB broth at 25°C were the control.

### Quantitative Real-Time PCR

The qPCR analysis was performed as we previously described (Li X. et al., 2019), and the experimental details are described in the Supporting Information.

### Antibacterial Activity of the Ethyl Acetate Extract of *Pseudomonas fluorescens* PF08

The growth inhibition of *S. aureus* and *A. salmonicida* CS12 was tested using the Oxford cup assay as previously described (Diao et al., 2014). One milliliter of overnight culture of *P. fluorescens* PF08 was inoculated to 100 mL of fresh LB broth and incubated at 28°C for 24 h. After centrifugation at 8000 × *g* for 10 min, the supernatant was collected. Metabolites were extracted using 100 mL of ethyl acetate. The organic phase was evaporated in a rotary evaporator and redissolved in 1 mL of 50% (v/v) methanol for further assay.

To prepare the test plates, the overnight cultures of *S. aureus* or *A. salmonicida* CS12 were diluted 1:100 in 20 mL of LB nutrient agar. Agar was poured into two plates containing autoclaved Oxford cups. Once the medium had solidified, 200 μL of ethyl acetate extract of *P. fluorescens* PF08 was added to the wells, and the plates were incubated for 24 h at 28°C. The antibacterial activity of the samples was evaluated by observing the growth inhibition zone. A control test was performed using 200 μL of 50% (v/v) methanol.

### Statistical Analysis

Data with normal distribution were analyzed by one-way analysis of variance (ANOVA) with *t*-test using the IBM SPSS Statistics program (version 20.0). The qPCR tests were performed for three times and comparisons of distributions between mean values of different groups were made by Duncan’s test at a statistical significance level of *p* < 0.05. Graphs were prepared in Graphpad Prism (version 8.02).

## Results and Discussion

### Genome Features of *Pseudomonas fluorescens* PF08

**FIGURE 1:**
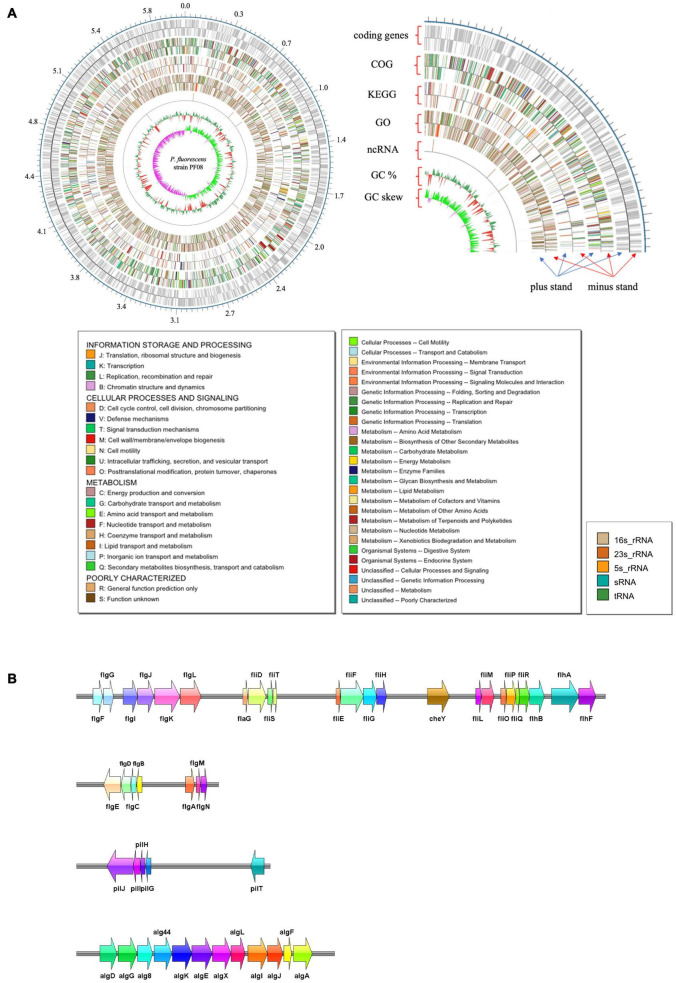
Graphical map of the completed chromosome of *Pseudomonas fluorescens* strain PF08. Numbers represent megabases (Mb). The type shown on the legend is the type that appears in the annotation result. The color of the box on the left corresponds to the color on the map **(A)**. The spoilage-related gene clusters in the genome of *P. fluorescens* strain PF08 **(B)**.

**FIGURE 2:**
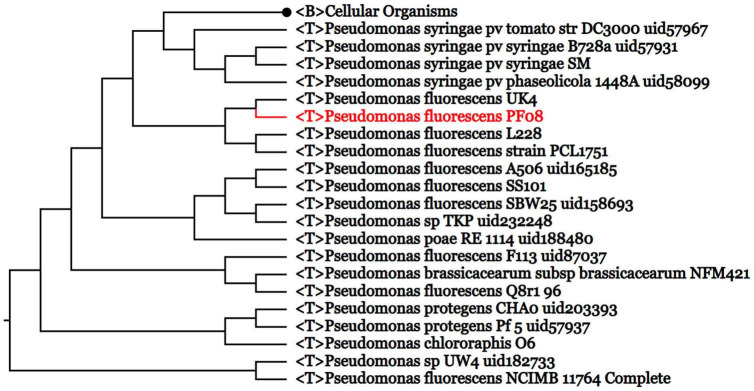
Genome phylogenetic tree based on the whole genome sequences of PF08 and some *Pseudomonas* strains compared in the study.

**FIGURE 3:**
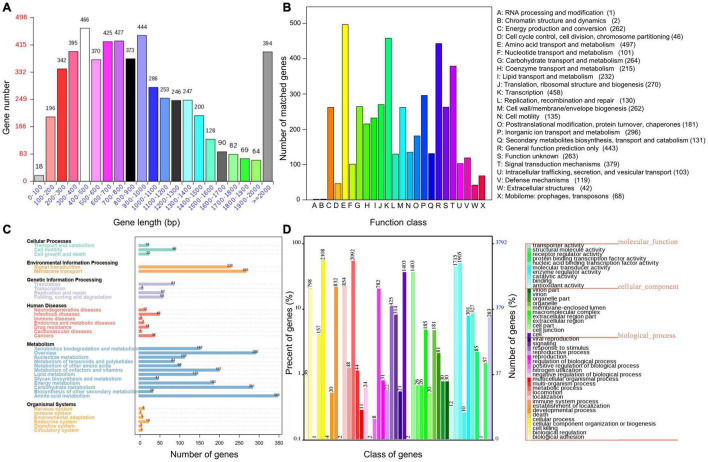
Distribution of gene sizes in *Pseudomonas fluorescens* PF08 **(A)** and the functional categories annotated using COG **(B)**, KEGG **(C)**, and GO **(D)**.

The genome of *P. fluorescens* PF08 is composed of a 6,030,573 bp chromosome (Figure 1A) with a G + C content of 60.7%. The genome analysis predicted a total of 5,600 genes consisting of 5,514 coding sequences. Genome phylogenetic tree based on the whole genome sequences of PF08 was performed and the result was shown in Figure 2. Further predicted were 66 tRNA genes, 16 rRNA genes, 4 ncRNA genes, and 164 pseudo genes. The size of the coding genes generally ranged from 200 to 1500 bp (Figure 3A). These features were similar to those of other *P. fluorescens* strains (Table 1), indicating that PF08 conformed to the basic features of *P. fluorescens*. Furthermore, PF08 was found to contain 69 tandem repeat sequences and 8 gene islands with an average length of 20,743 bp. Among the predicted genes, approximately 11 genes were related to mobile elements predicted as transposons and phage. Three CRISPR locations were also identified. The alignments of the genome sequences of *P. fluorescens* PF08 to those of *P. fluorescens* UK4 was performed using the Mauve program because of their close relationship based on the 16S rRNA genes. The alignments of some type strains of other *Pseudomonads*, including *P. fluorescens* UK4, *P. aeruginosa* PA01, and *P. putida* NBRC 14164, were also analyzed as control. As shown in Supplementary Figure 1, the genome sequences of *P. fluorescens* PF08 and *P. fluorescens* UK4 showed overall similarity. But the translocations between some fragments suggested differences in their metabolic patterns.

**TABLE 1:** General features of PF08 and other *Pseudomonas fluorescens* genomes.

Strains	Genome size	G + C Content	Total genes	CDS numbers	rRNAs	tRNAs	ncRNAs	Pseudo genes
*P. fluorescens* PF08	6030573 bp	60.67%	5600	5514	16	66	4	164
*P. fluorescens* UK4	6064456 bp	60.13%	5299	5178	19	68	1	22
*P. fluorescens* L228	6175426 bp	60.84%	5086	5732	7	63	4	135
*P. fluorescens* PCL1751	6143950 bp	60.36%	5684	5591	19	70	4	74

To reveal more comprehensive genetic information of PF08, the genome was functionally annotated using the COG, GO, and KEGG databases. The results are shown in Figure 3. COG clustering showed that *P. fluorescens* PF-08 had more coding sequences involved in amino acid transport and metabolism, transcription, general function prediction, signal transduction mechanism, and lipid transport, with 497, 458, 443, 379, and 232 sequences, respectively (Figure 3B). The genes of *P. fluorescens* PF-08 mainly comprised functions of amino acid metabolism (344), carbohydrate metabolism (281), signal transduction (226), and transmembrane transport (265) (Figure 3C). The knowledge that the bacterial signal transduction process can be directly involved in the QS system indicates that PF-08 has a potential for QS signaling molecular transduction.

The annotation information obtained by comparing PF08 genome with the GO database using IPRscan revealed that the functions of the genes in *P. fluorescens* PF-08 were mainly distributed in the categories of cellular process, metabolic process, catalytic activity, and binding. The numbers of annotations were 2108, 2092, 1905, and 1715, accounting for 12.55, 12.46, 11.35, and 10.21% of the total number of annotations, respectively. The findings indicate that strain PF-08 has pronounced activities of biometabolism, enzyme catalysis, and functional factor binding (Figure 3D).

### Gene Function Analysis

#### Quorum Sensing System

The LuxI/R type system, which is mainly composed of AHLs LuxI and specific receptor protein LuxR, is one of the most typical QS regulatory systems. The system has been found in many Gram-negative bacteria, including *Burkholderia*, *Escherichia coli, Hafnia alvei*, and *Pseudoalteromonas* (Weiss et al., 2008; Choudhary et al., 2013; Yu et al., 2019; Zhu et al., 2019). In this system, sufficient LuxI-secreted AHLs can interact with LuxR to form a complex that combines with the promoters of the downstream target genes to regulate their expression. This mediates the production of spoilage-required factors, including a variety of proteases (Galloway et al., 2011). Presently, the LuxI/R type system RhlI/R was detected in an organized operon in the genome of *P. fluorescens* PF08. In this system, *rhlI* encodes the AHLs synthetase, and *rhlR* encodes the response regulator. The RhlI/R system is reported to work as part of a cascade QS regulatory system to regulate the production of pyocyanin and chitinase in conjunction with the LasI/R system, in which the expression of *rhlI/R* is also positively regulated by the LasI/R system (Defoirdt, 2018). Interestingly, in this study, the genes encoding LasI/R were not found in the genome of *P. fluorescens* PF08, suggesting that the QS system RhlI/R may act as an independent system to accomplish the regulation of related factors, which is uncommon. This is consistent with previous observations (Steindler et al., 2009), who found that the two QS systems are not hierarchically organized and are both important for the colonization of *P. aeruginosa* on the rice rhizosphere.

During evolution, some Gram-negative bacteria have lost one or more LuxI-type AHL synthetases and have retained only unpaired LuxR-type regulator proteins. These proteins, termed LuxR solos or Orphan proteins, are still able to sense AHLs and regulate bacterial behavior accordingly. Thus, they have important roles in bacterial eavesdropping and inter- and intraspecific signaling (Hudaiberdiev et al., 2015). Therefore, exploring the *luxR* solo genes in the genome is valuable to understand the QS properties of *P. fluorescens* PF08. Eleven genes encoding LuxR family regulatory proteins were identified in the genome of PF08 in this study (Table 2). The response regulators PprA and PprB were identified as a two-component regulatory system of *P. aeruginosa* with the function to regulate virulence factor production and cell motility. Furthermore, some QS regulatory genes in *P. aeruginosa*, such as the *las* system and *rhl* system, are also mediated by this system, and most PprB-activated genes are activated by C_4_-HSL (Dong et al., 2005). In the *P. fluorescens* PF08 genome, only a separate gene, *pprB*, encoding the regulator PprB was identified. It was annotated as a *luxR* solo type regulator in the NR database. The presence of *pprB* explains the greater sensitivity of PF08 to C_4_-HSL than to other AHLs, which we previously demonstrated (Li T. et al., 2019). The hybrid histidine kinase RcsC and the response regulator RcsB are homologous to environmentally responsive two-component regulators capable of promoting biofilm formation and the appearance of fimbriae (Stout and Gottesman, 1990; Nicastro et al., 2009). In this study, the *rcsB* gene was identified as a *luxR* solo type regulator in the genome of PF08, which is helpful to further understand the regulation process of biofilm formation in PF08. The *sdiA* gene predicted to encode LuxR homologs to regulate the ftsQAZ locus and virulence factors EspD in *E. coli* and *Salmonella enterica* has also been identified in PF08 (Kanamaru et al., 2000). In addition, some genes encoding putative LuxR solo type regulator, such as the environmental sensing gene *agmR* and the virulence regulator gene *bvgA*, were also annotated in the PF08 genome. These findings could genetically explain the ability of PF08 to enhance its virulence and spoilage factors through AHLs eavesdropping (Li T. et al., 2019).

**TABLE 2:** Genes encoding predicted LuxR solo type proteins in the genome of *Pseudomonas fluorescens* PF08.

Genes	Location in Chromosome (bp)	Size (bp)	Annotation in NR database
*pprB*	690335–691084**^+^**	749	LuxR family transcriptional regulator
*malT*	1053557–1056097**^+^**	2540	LuxR-type DNA-dependent transcriptional regulator
*rcsB*	1220206–1220844**^–^**	638	Captular synthesis response regulator
*csgD*	1963811–1964122**^+^**	311	LuxR- type DNA-binding transcriptional regulator
*evgA*	2305174–2305800**^+^**	626	putative LuxR family regulatory protein
*narL*	2464863–2465465**^–^**	602	LuxR family transcriptional regulator
*uhpA*	3693204–3693830**^–^**	626	LuxR family Transcriptional activator protein
*fixJ*	3729441–3730937**^+^**	1496	Sensory box transcriptional regulator, LuxR family protein
*bvgA*	3949169–3949795**^+^**	626	LuxR family transcriptional regulator
*agmR*	4717227–4717874**^–^**	647	LuxR family DNA-binding response regulator
*sdiA*	5184018–5184668^+^	650	LuxR family transcriptional regulator

*“+” means the transcription begins from 5′.*

#### Motility and Biofilm Formation

Flagellar driven motility is an effective way for bacteria to escape immune system components and fungicides. The motility is mainly activated by the QS system under environmental stress (Díaz et al., 2011). Pili can mediate the adhesion of bacteria to different surfaces, which is essential to initiate biofilm formation. Thus, pili pose risks in medical facilities and the food industry. Our preliminary study observed the good motility and biofilm formation ability of *P. fluorescens* (Li et al., 2018). Presently, we explored the genes associated with biofilm formation and motility of *P. fluorescens* PF08. A total of 34 genes related to flagella were identified. Of these, 13 belonged to the *flg* gene cluster located in 3 flagellar operons. These genes regulate the synthesis of flagella by sensing the integrity of the flagellar structure (Chilcott and Hughes, 2000). Gene clusters including *fliD/S/T/E/F/G/H* and *fliL/M/O/P/Q/R* encode the flagellar assembly proteins. These clusters were detected in *P. fluorescens* PF08 (Figure 1B). The FliF protein forms the M-ring embedded in the cytoplasmic membrane of the basal part of the flagellar. The integral membrane proteins located in the MS-C loop (FlhA, FlhB, FliP, FliQ, FliR, and FliO) and soluble proteins (FliL, FliH, and FliJ) form the output device of flagella. FlhA and FlhB proteins comprise the platform for the attachment of FliL and FliH. The corresponding response of bacteria to external environmental changes is called chemotaxis. Chemotaxis is regulated by a two-component system involving the methyl-accepting chemotaxis protein (MCP). MCP can sense the changes of chemical signals in the external environment and adjusts the motility of bacterial flagella by regulating the two-component system (Kulasekara et al., 2013). The histidine kinase CheA and response regulator CheY that perform signal transduction are important components of the two-component regulatory system. The genes encoding CheA and CheY were detected in *P. fluorescens* PF08 in this study, indicating that the bacteria can adapt to environmental changes.

Concerning biofilm establishment, gene clusters involved in the biosynthesis of type IV pili were detected in the *P. fluorescens* PF08 genome in this study. The genes can promote bacterial adhesion and biofilm formation. Some of these genes are highly conserved in *Pseudomonas* sp. Two examples are *pilG* and *pilJ*, which are required for pili extension and twitching motility (Darzins, 1993; DeLange et al., 2007). Cyclic bis (3′–5′) diguanylic acid (c-di-GMP) is a second messenger molecule commonly found in bacteria. c-di-GMP improves the synthesis of cellulose, a major biofilm component, and therefore plays an important role in regulating biofilm formation (Tischler and Camilli, 2004). Genes encoding the key enzymes in the synthesis and metabolism of c-di-GMP were detected in this study. These enzymes included diguanylate cyclase (GGDEF domain), c-di-GMP synthetase, and phosphodiesterase (EAL domain). Their presence suggests the existence of the c-di-GMP regulatory pathway in *P. fluorescens* PF08. Alginate and certain extracellular proteins form the reticular skeleton that maintains the mechanical stability of biofilms. These components are crucial for the extracellular polymer (Laue et al., 2006). In the initial stage of extracellular polymer formation, a high content of alginate is important in the attachment of bacteria to the surface. The presence of a series of alginate synthesis genes, including *alg8, alg44*, *algA*, *algK*, *algE*, *algQ*, and *algX*, and the aforementioned genes explains the strong biofilm formation and motility ability of *P. fluorescens* at the genetic level, which was confirmed in our previous study (Li et al., 2018).

#### Protease and Lipase Production and Typical Metabolic Pathways Involved in Spoilage

Extracellular proteases secreted by spoilage bacteria can decompose proteins in food matrix into small nitrogen-containing substances to maintain their own growth, accompanied by rapid deterioration of food quality including taste and flavor. Protease is the most important spoilage factor. Therefore, knowledge of protease-related genes is necessary to better understand and inhibit food spoilage caused by *P. fluorescens* PF08. Presently, a total of 13 protease-encoding genes, including the metalloprotease *aprX*, alkaline protease *aprA*, ATP-dependent Clp protease *clpP*, Zn-dependent metalloprotease *sprT*, aspartyl protease *asP1*, and serine protease *serP*, were identified in the PF08 genome. Among these, alkaline proteases are the most important type of proteases that cause food spoilage. Scatamburlo et al. (2015) demonstrated that the alkaline proteases from *Pseudomonas* sp. cause the spoilage of goat milk. Similarly, bacterial metalloproteases have been implicated in the spoilage of diverse foods. Reducing the activity of metalloprotease AprX can improve the quality of ultra-high temperature-treated milk during storage (Zhang et al., 2019). Furthermore, the serine protease SerP in *H. alvei* H4 may be crucial in the spoilage of instant sea cucumber (Zhu et al., 2020). In addition to these typical proteases, other genes encode some spoilage-related lipases in PF08, such as lipase D (*lipD*), phospholipase (*rssA*), lysophospholipase (*pldB*), and triacylglycerol esterase (*estA*). These lipases can degrade fats and lipoproteins of aquatic products to produce free fatty acids, glycerol, and choline, thus accelerating spoilage (Glantz et al., 2020). Choline can be further converted into harmful end products like dimethylamine and trimethylamine, which detrimentally affect the flavor of aquatic products. The foregoing genes found in the *P. fluorescens* PF08 genome confirmed the spoilage potential of the bacterium from a genetic perspective.

Some metabolic pathways in bacteria are responsible for further metabolizing proteins, carbohydrates, and lipids. Various substances produced by their degradation form volatile components, such as trimethylamine, putrescine, and hydrogen sulfide. These volatiles are associated with putrid off-odors in aquatic products and are markers of spoilage. Pathways of amino acid metabolism, sulfur metabolism, and amine metabolism are therefore considered to be involved in food spoilage. The genetic information related to these relevant metabolic pathways was presently analyzed to explain the spoilage potential of *P. fluorescens* PF08. As shown in Figure 3C, amino acid metabolism was the primary function of *P. fluorescens* PF08 in KEGG annotations, suggesting good potential for protein degradation. The *Cyc* gene cluster is an important gene cluster involved in sulfur metabolism. Genes in the cluster encode key enzymes, including adenylylsulfate kinase CysC and phosphoadenosine phosphosulfate reductase CysH. These enzymes catalyze the multi-step reduction of adenosine-5’-phosphosulfate to hydrogen sulfide in PF08. Other genes associated with sulfur metabolism that were detected in the PF08 genome included *glpE* (encoding thiosulfate sulfurtransferase), *ssuD* (encoding alkanesulfonate monooxygenase), and *sseA* (encoding 3-mercaptopyruvate sulfurtransferase). Their presence is indicative of sulfur metabolic activity of PF08.

Amine metabolism of SSOs is another important metabolic pathway involved in the spoilage of aquatic products. This pathway was also analyzed in this study. Putrescine is one of the main causes of the unpleasant smell of rotten aquatic products. The genes encoding a series of proteins responsible for the production of putrescine, such as putrescine transport system permease protein, putrescine-binding periplasmic protein E, putrescine aminotransferase, and gamma-glutamylputrescine oxidase, were identified in the ammonia metabolism pathway of *P. fluorescens* PF08. Genes encoding trimethylamine dehydrogenase and spermidine synthase were also detected. These enzymes are involved in trimethylamine metabolism and spermidine production, respectively. The results demonstrate that PF08 has the potential for amine metabolism. Interestingly, although Gloria et al. (2011) detected cadaverine and histamine in spoilage milk inoculated with *P. fluorescens*; however, no genes related to cadaverine and histamine production were found in *P. fluorescens* PF08 in this study.

#### Environmental Stress Response

The continuous consumption of proteins and ions caused by microbial metabolism leads to the irreversible deterioration of the food microenvironment. To adapt to this change of the environment, microorganisms adjust their metabolic activities to maintain their normal growth by secreting some proteins involved in homeostasis. We have demonstrated that *P. fluorescens* PF08 is the SSO of turbot during low temperature storage, suggesting that this bacterium has a particular advantage in interbacterial competition during this process (Li T. et al., 2019). Investigation of genes related to the stress response in the *P. fluorescens* PF08 genome is helpful to reveal the reasons behind this advantage. RpoS and RelA were detected in the genome. They are considered typical environmental response regulators involved in stress survival in bacteria. Liu et al. (2018) found the RpoS-deficient mutant strains of *P. fluorescens* were less tolerant to environmental factors of high temperature, low pH, and starvation. The QS system of the mutant strains was also suppressed. In *P. fluorescens* PF08, the *rpoS* gene may play a similar role. Pyoverdine is a low molecular weight organic compound that can bind Fe^2+^ efficiently under low iron stress conditions to maintain the necessary Fe^2+^ uptake in cells. The synthesis of pyoverdine in bacteria is also regulated by the QS system (Xu et al., 2019). The *pvdA* and *pvdE* genes regulate the biosynthesis of pyoverdine. The presence of both genes in PF08 indicate that PF08 may have a certain growth advantage in low iron stress. Other genes identified in PF08 included some general stress response-related genes, including the antioxidant protein-coding gene *ahpC*, glutathione peroxidase-coding gene *bsaA*, starvation stringent protein-coding genes *sspA* and *sspB*, and heavy metal detoxification-related genes *cusS* and *merR*. The existence of these genes may be a potential reason for the environmental adaptability and competitive advantage of PF08.

#### Drug Resistance and Antimicrobial Metabolites

The accumulation of antibiotics in the environment and animal food farming processes imposes antibiotic pressure on bacteria, which had driven the evolution of a series of resistance pathways. Bacterial drug resistance can be transmitted by conjugation transfer, which poses a great challenge to food safety. More than 40 drug resistance-related genes were found in the genome of PF08. Hydrolysis of antibiotics into harmless metabolites by secretion of some key enzymes is the main pathway of bacterial drug resistance. β-lactamase and penicillin amidase encoded in PF08 can accelerate the metabolism of intracellular penicillin, thereby enhancing the tolerance of PF08 to penicillin. The gene encoding D-amino-acid oxidase (DAO), that can degrade cephalosporin, was also detected in PF08. In addition, the rapid efflux through the transport system of antibiotics that are not degraded is another effective means of bacterial survival. A total of 12 ABC-type multidrug transport system-related genes and 24 multidrug efflux pump genes were identified in the PF08 genome. The system composed of these coding genes can efficiently recognize specific lipophilic antibiotics and transport them out of the cells. The discovery of these genes has contributed to a better understanding of the drug resistance of PF08.

The antibacterial properties of the metabolites of PF08 were also investigated. This antibacterial property was implied by the observed advantage of PF08 in competing with other bacteria for survival during turbot spoilage. As shown in Supplementary Figure 2, the ethyl acetate extract of PF08 produced significant zones of growth inhibition in plates inoculated with *S. aureus* and *A. salmonicida*. The findings indicated the bacteriostasis of metabolites by PF08. Indole alkaloids are one of the marine alkaloids. They are derived mainly from marine organisms, such as sponges and mosses, which have significant antibacterial, antitumor, and other biological activities. The genes involving in the biosynthesis of indole alkaloids were detected in PF08, indicating the potential of PF08 to synthesize alkaloids. Polyketides are natural compounds produced by bacteria and fungi. The various polyketides include macrolides, tetracyclines, and polyethers. These compounds have antibacterial and anti-infection activities. In bacteria, the biosynthesis of polyketides is regulated by polyketide synthase. Construction of the carbocyclic scaffold catalyzed by polyketide cyclase is a key step in the biosynthesis. The genes encoding polyketide synthase and polyketide cyclase were detected in PF08, as were antibiotic biosynthesis monooxygenase (ABM) superfamily genes. Qin et al. (2019) identified the non-catalytic role of ABM superfamily proteins in polyketide synthesis in maintaining the fidelity of the biosynthetic pathway. The results suggest that PF08 may have the ability to secrete this kind of antibacterial substance. These antimicrobial substances produced by PF08 are mostly lipophilic and can be transported extracellularly using their own drug efflux pump system to reduce intracellular accumulation and thus inhibit the growth of competing microorganisms without damaging themselves.

### Quorum Sensing System Regulation of Transcription of Spoilage-Related Factors in PF08

**FIGURE 4:**
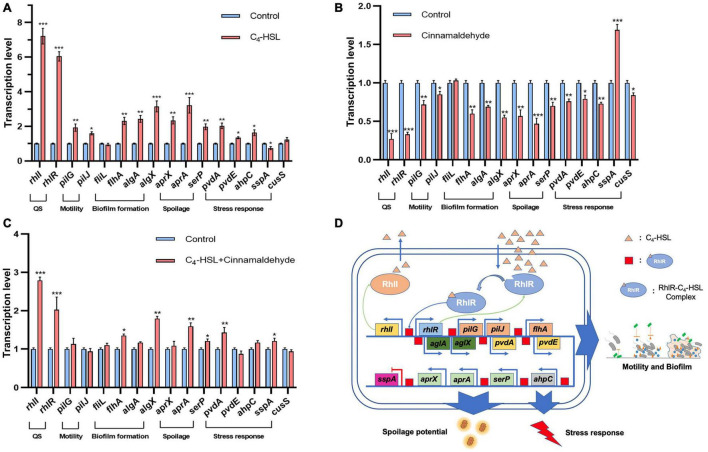
Transcription levels of typical genes related to spoilage and stress response in *Pseudomonas fluorescens* PF08 after incubation with 20 μg/mL C_4_-HSL **(A)**, or 0.1 μL/mL cinnamaldehyde **(B)**, or 20 μg/mL C4-HSL and 0.1 μL/mL cinnamaldehyde **(C)** until an optical density at 595 nm (OD 595) of 1 was reached. Schematic diagram of the regulation of QS system in PF08 on the spoilage potential **(D)**. Data are presented as means ± SD (*n* = 3, *P < 0.05, **P < 0.01, ***P < 0.001).

The QS system has been widely reported as a key factor in the regulation of food spoilage (Liu et al., 2018). However, the role of QS system in the regulation of spoilage-related genes in *P. fluorescens* has not been systematically reported. Therefore, the effect of C_4_-HSL, the typical AHL synthesized by the RhlI protein, on the transcription of *rhlI/R* and other typical spoilage-related genes, including *pilG*, *pilJ*, *fliL, flhA, algA, algX, aprX*, *aprA*, *serP, pvdA, pvdE, ahpC, sspA*, and *cusS*, in PF08 was further analyzed in this study to explore the regulatory role of QS system on the spoilage potential of PF08. The results are presented in Figure 4A. As an agonist of the QS system, C_4_-HSL significantly increased the transcription of *rhlI/R* genes in PF08, suggesting that the QS system was successfully activated because of the feedback regulation mechanism of the Lux-type QS system. Transcription of some genes related to motility and biofilm formation, such as *pilG*, *pilJ*, *flhA, algA*, and *algX*, was increased with the addition of C_4_-HSL. More importantly, the transcription of genes, including *aprX*, *aprA*, and *serP*, which encode proteases that are active in spoilage was all promoted after incubating with C_4_-HSL. The findings suggest, at the genetic level, the regulatory role of QS on the spoilage potential of PF08. The addition of C_4_-HSLs also promoted the transcription of *pvdA, pvdE, ahpC*, and *cusS* related to stress response. This might make PF08 more competitive than other bacteria during turbot spoilage. Interestingly, the transcription of *sspA* that encodes starvation stringent protein was inhibited after adding C_4_-HSL. These results suggested that the addition of C_4_-HSL put the bacteria in a more “excited” state.

Although we found that the transcription of some key spoilage related genes changed synchronously with the promotion of QS system. However, this is not enough to demonstrate their correlation with QS system. The QS inhibitory effect of cinnamaldehyde on *Pseudomonas* has been wildly reported (Topa et al., 2020). Our previous study also showed that cinnamaldehyde can inhibit the QS system of *P. fluorescens* by targeting LuxR-type QS receptor without influencing AHLs (Li et al., 2018). Therefore, cinnamaldehyde was used as a QS inhibitor here and the transcription of these key genes in cinnamaldehyde treated PF08 was further analyzed. As shown in Figure 4B, the downregulation of the transcription of *rhlI* and *rhlR* gene proved the inhibition of the QS system in PF08 caused by cinnamaldehyde. Moreover, the transcription of *pilG*, *pilJ*, *flhA*, *algA*, *algX*, *aprX*, *aprA*, *pvdA*, *pvdE*, *ahpC*, and *cusS* were all down-regulated by cinnamaldehyde. Correspondingly, the transcription level of *sspA* increased after the treatment of cinnamaldehyde. More importantly, when cinnamaldehyde and C_4_-HSL were added simultaneously, the transcription promotion of these key genes caused by C_4_-HSL was significantly weakened (Figure 4C). The results demonstrate the correlation between the transcription of spoilage related genes and the QS system (*rhlI/R*) in PF08. Although knockout of the QS system may be a more direct method, since C_4_-HSL and cinnamaldehyde have been proved to act directly on the QS system, we believe that the spoilage potential of PF08 is likely to be regulated by QS system by regulating the transcription of relevant genes (Figure 4D).

### Effects of Different Conditions on the Transcription of *rhlI/R* System in PF08

**FIGURE 5:**
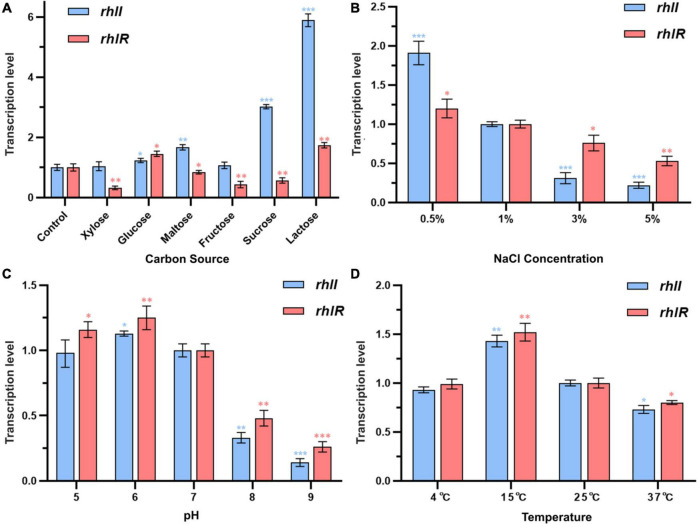
Transcription levels of *rhlI/R* genes in *Pseudomonas fluorescens* PF08 after incubation in different conditions until an optical density at 595 nm (OD 595) of 1 was reached. **(A)** Different carbon sources in culture medium; **(B)** different NaCl concentrations of the culture medium; **(C)** different pH of the culture medium; **(D)** different temperatures during incubation. Data are presented as means ± SD (*n* = 3, *P < 0.05, ^**^P < 0.01, ^***^P < 0.001).

Specific spoilage organisms mainly regulate the production of spoilage-required factors, such as protease, by the QS system, which can sense environmental changes during food spoilage and provides feedback by initiating the transcription of relevant genes. Therefore, the growth environment may change the characteristics of bacteria and may further affect their spoilage potential by stimulating the QS system. The effects of different environmental conditions on the transcription of *rhlI* and *rhlR* genes in PF08 were evaluated using qPCR. As shown in Figure 5, the environmental factors could affect the transcription of *rhlI* and *rhlR* in PF08. Carbon is an essential and basic nutrient for microbial growth. The carbon source can be a potential factor affecting the transcription of QS systems owing to the different metabolic pathways of bacteria for the different sources. As shown in Figure 5A, the relative transcription of *rhlI* was the highest during culture with lactose as the carbon source. Growth was 5.6-fold higher than that of the control group. The relative transcription of *rhlI* was the lowest when xylose was the carbon source. The transcription of *rhlI* decreased sequentially when the carbon sources were sucrose, maltose, glucose, and fructose. Similar to the *rhlI* gene, the relative transcription of *rhlR* was the highest and lowest when lactose and xylose were the respective carbon sources. However, the effect of carbon source on *rhlR* transcription was not significant compared with that on *rhlI* transcription and was mostly inhibitory.

Figure 5B depicts the transcription of *rhlI* and *rhlR* genes in PF08 cultured in different NaCl concentrations. The transcription of the *rhlI* and *rhlR* genes decreased significantly with increasing NaCl concentration and was the lowest when the NaCl concentration was 5% (w/v). The transcription of *rhlI* and *rhlR* was only 22 and 53% of that apparent at 1% NaCl, respectively. In contrast, the transcription of *rhlI* and *rhlR* increased when the NaCl concentration < 1%. The respective transcription was 1.9 and 1.2-times higher, respectively, than the expression at 1% NaCl. The results showed that the concentration of NaCl in the culture environment could affect the transcription of *rhlI* and *rhlR* genes in *P. fluorescens*, and that a low NaCl concentration was more beneficial to the transcription of the two genes. Similarly, Yarwood and Schlievert (2003) showed that the secretion of microbial AHLs is susceptible to stress from low or high NaCl concentrations in the environment.

The transcription of *rhlI* and *rhlR* genes in PF08 cultured in different pH conditions is shown in Figure 5C. An acidic environment had little effect on the transcription of *rhlI* and *rhlR* genes. However, when the culture environment was alkaline, *rhlI* and *rhlR* transcription was significantly inhibited. When the initial pH of the medium was 9, the transcription of *rhlI* and *rhlR* genes in PF08 had the lowest values, which were 14 and 16% of those at pH 7. Our previous study demonstrated that the secretion of AHLs from *P. fluorescens* was significantly reduced in alkaline environments. We suggested that this reflects the destruction of the lactone ring of AHLs in alkaline environments, resulting in a reduction in their chemical stability (Yang et al., 2017). The results obtained here demonstrate that this may be due to the synergistic inhibition of the transcription of AHLs synthetase gene *rhlI* under alkaline conditions.

Temperature is another important environmental factor affecting the growth and metabolism of bacteria. Previous studies have shown that the bacterial QS system is affected by temperature. Therefore, the effects of culture temperature on the transcription of *rhlI* and *rhlR* genes in PF08 were explored. The results are shown in Figure 5D. When the culture temperature was 15°C, the relative transcription levels of *rhlI* and *rhlR* genes were the highest (1.43 and 1.52-times higher, respectively, than those at 25°C). In contrast, the relative transcription levels of *rhlI* and *rhlR* genes in PF08 cultured at 37°C were only 73 and 80% of the values at 25°C. This is consistent with the observations of Zhu et al. (2020), who found that the culture temperature could affect the expression of *luxI/R* genes in *H. alvei* H4, with expression suppressed at high temperatures. Interestingly, the relative transcription of *rhlI* and *rhlR* genes remained essentially unchanged when incubated at 4°C, reaching 93 and 99% of the values at 25°C. This may reflect that *P. fluorescens* is a psychrotrophic bacterium. The results suggests that PF08 may still effectively regulate the production of spoilage-required factors through the QS system at the storage temperature of aquatic products (4°C).

## Conclusion

The findings expand the knowledge of genes associated with QS system and spoilage potential, including protease production, biofilm formation, and sulfur and amine metabolism, in the genome of *P. fluorescens* PF08. Furthermore, the regulatory effect of the QS system on the transcription of typical genes related to the spoilage potential of PF08 was demonstrated. The influence of environmental factors on the transcription level of the *rhlI/R* system in PF08 was evaluated. The results further clarify the relationship between QS system and the spoilage potential of PF08 and will inform the development of QS-targeted spoilage control strategies.

## Data Availability Statement

The datasets presented in this study can be found in online repositories. The names of the repository/repositories and accession number(s) can be found in the article/Supplementary Material.

## Author Contributions

DW conceived and designed the experiments, analyzed the data, contributed reagents, materials, and analysis tools, authored or reviewed drafts of the manuscript, and approved the final draft. FC conceived and designed the experiments. LR analyzed the data, contributed reagents, materials, and analysis tools, prepared the figures and/or tables. XT, XL, and QL conceived and designed the experiments. JL conceived and designed the experiments, approved the final draft, had overall responsibility for this project. TL conceived and designed the experiments, performed the experiments, analyzed the data, prepared the figures, and/or tables, authored or reviewed drafts of the manuscript. All the authors contributed to the article and approved the submitted version.

## Conflict of Interest

The authors declare that the research was conducted in the absence of any commercial or financial relationships that could be construed as a potential conflict of interest.

## Publisher’s Note

All claims expressed in this article are solely those of the authors and do not necessarily represent those of their affiliated organizations, or those of the publisher, the editors and the reviewers. Any product that may be evaluated in this article, or claim that may be made by its manufacturer, is not guaranteed or endorsed by the publisher.

## Footnotes

1
https://www.ncbi.nlm.nih.gov/genome

2
http://www.repeatmasker.org/

## Supplementary Material

Supplementary Figure 1 Alignment of the genomes of *Pseudomonas fluorescens* PF08 and different type strains of other *Pseudomonas*. *P. fluorescens* UK4 **(A)**, *P. aeruginosa* PA01 **(B)**, *P. putida* NBRC 14164 **(C)**, *P. syringe* BIM B-268 **(D)**. The same color represents the colinear regions of similarity.

Supplementary Figure 2 The inhibitory effect of ethyl acetate extract of *Pseudomonas fluorescens* PF08 on the growth of *Staphylococcus aureus* and *Aeromonas salmonicida* CS12. (a) Metabolites of PF08; (b) Equal amount of 50% (v/v) methanol used as control.
