# Supplementary material for: Complete Genome Analysis Reveals the Quorum Sensing-Related Spoilage Potential of Pseudomonas fluorescens PF08, a Specific Spoilage Organism of Turbot (Scophthalmus maximus)
Source: Front Microbiol. 2022 Apr 18;13:856802. doi: 10.3389/fmicb.2022.856802 (PMC9062736; doi:10.3389/fmicb.2022.856802)
Supplement: Supplementary Figure 1 — Alignment of the genomes of Pseudomonas fluorescens PF08 and different type strains of other Pseudomonas. P. fluorescens UK4 (A), P. aeruginosa PA01 (B), P. putida NBRC 14164 (C), P. syringe BIM B-268 (D). The same color represents the colinear regions of similarity. [file Data_Sheet_1.docx]

**Supporting Information**

**Complete genome analysis reveals the quorum sensing-related spoilage potential of *Pseudomonas fluorescens* PF08, a specific spoilage organism of turbot (*Scophthalmus maximus*)**

**Dangfeng Wang^a, b^, Fangchao Cui^b^, Likun Ren^c^, Xiqian Tan^b^, Xinran Lv^b^, Qiuying Li^b^, Jianrong Li^a^*^, b^,** **Tingting Li^d^***

aCollege of Food Science and Technology, Jiangnan University, Wuxi Jiangsu, 214122, China

bCollege of Food Science and Technology, Bohai University; National & Local Joint Engineering Research Center of Storage, Processing and Safety Control Technology for Fresh Agricultural and Aquatic Products, Jinzhou Liaoning, 121013, China

cBeijing Key Laboratory of Flavor Chemistry, Beijing Technology and Business University (BTBU), Beijing, 100048, China

dKey Laboratory of Biotechnology and Bioresources Utilization, Dalian Minzu University, Ministry of Education, Dalian Liaoning, 116029, China

eKey Laboratory of Food Science and Engineering of Heilongjiang Province, College of Food Engineering, Harbin University of Commerce, Harbin Heilongjiang, 150076, China

**Genome sequencing and assembly**

The genomic DNA (gDNA) of *P. fluorescens* PF08 (10 mL) was extracted and purified with TaKaRa^®^ Bacteria DNAiso Reagent Kit (TaKaRa Bio, Shiga, Japan) following the manufacturer’s instructions. The harvested gDNA was detected using 1% agarose gel electrophoresis and quantified using a NanoDrop Spectrophotometer (Thermo Fisher Scientific, Waltham, MA, USA). Sequencing was performed using a Paciﬁc Biosciences (PacBio) RS II instrument with P4-C2 chemistry. The genome of *P. fluorescens* PF08 was sheared to approximately 10 kbp using a g-TUBE (Covaris, Woburn, MA, USA) and was sequenced using Single Molecule, Real-Time (SMRT) technology. A total of 93,810 clean reads with a mean read length of approximately 10 kbp (N50 size of 16,356 bp), which corresponded to approximately 100× coverage, were obtained after filtering out the low-quality reads using SMRT 2.3.0 software (Berlin et al., 2015; Koren and Phillippy. 2015). Finally, the *de novo* assembly of filtered clean reads were performed to generate one contig without gaps using the Hierarchical Genome Assembly Process method based on the SMRT analysis. The data visualization of the circular genome of PF08 was performed using Circos software ver. 0.66 and in-house python scripts.

**Quantitative real-time PCR**

The total RNA of different samples was isolated with TRIzol reagent (Sigma-Aldrich) following the manufacturer’s instructions. The RevertAid First -Strand cDNA Synthesis Kit (#K1621; Thermo Fisher Scientific) was used to reverse transcribe the qualified RNA. The qPCR system (20 μL of reaction volume: 10 µL of 2×SYBR Green PCR Master Mix, 4 µL of Sterilizer ddH_2_O, 0.5 µL of F primer and R primer, 5 µL of cDNA) was prepared following the instructions of Power SYBR^®^ Green PCR Master Mix (Applied Biosystems^®^ Cat: 4367659; Thermo Fisher Scientific), and qPCR analysis was performed using the CFX Connect^™^ System (BIO-RAD Laboratories, Hercules, CA, USA), under the following conditions: 95 °C for 3 min, followed by 40 cycles of denaturation at 95 °C for 10 s, annealing at 55 °C for 20 s, and extension at 72 °C for 20 s. The housekeeping gene 16S rRNA was used as an internal reference. The melt curve was established in the range of 65–95 °C. The transcription levels of different genes were quantiﬁed with the following standard formula by calculating 2^-^*^△△^*^Ct^. The primers for qPCR are shown in Table S1. All experiments were performed three times (n = 3 per experiment) with the results reported as average ± standard deviation (SD).

| Table S1. Quantitative real-time PCR primers used in this study | | | |
| --- | --- | --- | --- |
| Primer | PCR product size (bp) | Oligonucleotide sequence (5′–3′) | Use |
| *rhlI* F1 | 168 | AAGTCTTCGGGTTTCTGTGC | objective gene |
| *rhlI* R1 |  | TCACTGCCACCACCTGATTA |  |
| *rhlR* F1 | 155 | GACCCCTTTTACCCGACCA | objective gene |
| *rhlR* R1 |  | CAAACAGTGCGTCGTTCCA |  |
| *pilG* F1 | 84 | GAAGAACGTGGGGTGCGAAGTC | objective gene |
| *pilG* R1 |  | ATAATGTGCGGGTGGTGGTCAAC |  |
| *pilJ* F1 | 102 | CCGTAGCCGTTCGCAGATCATC | objective gene |
| *pilJ* R1 |  | TCGTAGGTGGACTGGGTGTTGAG |  |
| *fliL* F1 | 82 | CAGAACGGTCGGCAACGCTAC | objective gene |
| *fliL* R1 |  | GCACCTTGAGGGCATCCATGTC |  |
| *flhA* F1 | 145 | GGCACCCTGACGGACCTCTC | objective gene |
| *flhA* R1 |  | GCAGCACGACGATGGACAAGG |  |
| *algA* F1 | 137 | CAGGTCAAGCACATCTCGGTCAAG | objective gene |
| *algA* R1 |  | TTCTCGCAGAGCAGGAACACATTC |  |
| *algX* F1 | 129 | CGCAACAGCCGTCATCAGGTC | objective gene |
| *algX* R1 |  | TGTCTCGGATGTTTCGGGTTTGTC |  |
| *aprX* F1 | 131 | CGGTATCAGCGATGAGCGTTATGG | objective gene |
| *aprX* R1 |  | GCTCCTGCAACACCACCACTG |  |
| *aprA* F1 | 138 | GTGAATCCACGCCGCAGAACC | objective gene |
| *aprA* R1 |  | TGCCTGGCTGCCTATTGAATTGAC |  |
| *serP* F1 | 128 | GGCTCGCTTACATCACAGGTCAC | objective gene |
| *serP* R1 |  | ACATTCAAGGTGCCGATGGAGTTG |  |
| *pvdA* F1 | 115 | ATCATCGGTGGCGGGCAGAG | objective gene |
| *pvdA* R1 |  | TGTCGTCGGCAGGCTTGAGG |  |
| *pvdE* F1 | 103 | TTCGTCGGTCAGAAGGTGATTGC | objective gene |
| *pvdE* R1 |  | TCAGGCGGTGAGTGCGGTAG |  |
| *ahpC* F1 | 108 | CACGAAGAGCACGGCGAAGTC | objective gene |
| *ahpC* R1 |  | CTTGCCAGCGTTCTCGGTCAG |  |
| *sspA* F1 | 134 | GCCTGGTGGATTGCTGCCTATTAC | objective gene |
| *sspA* R1 |  | CAGACTTGCCTGGAAAGCCTCAC |  |
| *cusS* F1 | 137 | TCGCTACTGGACCTGGTGATGG | objective gene |
| *cusS* R1 |  | GTCGGTCTGGCGGCAAAGTC |  |
| *16S rRNA* F1 | 133 | GCTTTCGCCCATTGTCCAA | reference gene |
| *16S rRNA* R1 |  | TGGTGGGGTAATGGCTCAC |  |

All primers for qPCR were designed with Primer 5.0 software and synthesized by Sagan (Shanghai, China)

Table. S2 Genes encoding spoilage-related proteins in the genome of *Pseudomonas fluorescens* PF08

| Behavior | Classification | Genes | Location in Chromosome (bp) | Size (bp) | Proteins Description |
| --- | --- | --- | --- | --- | --- |
| Motility and Biofilm Formation | Flagellar Related | *flhB* | 43027-43266+ | 239 | flagellar biosynthesis protein FlhB |
|  |  | *motA* | 54045-54776+ | 731 | flagellar motor protein MotA |
|  |  | *motB* | 54886-56409+ | 1523 | flagellar motor protein MotB |
|  |  | *cheA* | 1237599-1239905+ | 2306 bp | chemotaxis protein CheA |
|  |  | *flgF* | 1741247-1741993+ | 746 | flagellar basal body rod protein FlgF |
|  |  | *flgG* | 1742061-1742846+ | 785 | flagellar basal body rod protein FlgG |
|  |  | *flgI* | 1743595-1744710+ | 1115 | flagellar P-ring protein FlgI |
|  |  | *flgJ* | 1744715-1745977+ | 1262 | flagellar rod assembly protein FlgJ |
|  |  | *flgK* | 1746048-1748012+ | 1964 | flagellar hook protein FlgK |
|  |  | *flgL* | 1748027-1749646+ | 1619 | flagellar hook-associated protein FlgL |
|  |  | *flaG* | 1752902-1753246+ | 344 | flagellar protein FlaG |
|  |  | *fliD* | 1753330-1754694+ | 1364 | flagellar cap protein FliD |
|  |  | *fliS* | 1754832-1755212+ | 380 | flagellar biosynthesis protein FliS |
|  |  | *fliT* | 1755238-1755534+ | 296 | flagellar assembly protein FliT |
|  |  | *fliE* | 1760125-1760454+ | 329 | flagellar hook-basal body protein FliE |
|  |  | *fliF* | 1760471-1762246+ | 1775 | flagellar M-ring protein FliF |
|  |  | *fliG* | 1762239-1763258+ | 1019 | flagellar motor switch protein FliG |
|  |  | *fliH* | 1763269-1764033+ | 764 | flagellar assembly protein FliH |
|  |  | *cheY* | 1767198-1768910+ | 1712 | chemotaxis protein CheY |
|  |  | *fliL* | 1770958-1771404+ | 446 | flagellar basal body protein FliL |
|  |  | *fliM* | 1771414-1772382+ | 968 | flagellar motor switch protein FliM |
|  |  | *fliO* | 1772895-1773329+ | 434 | flagellar assembly protein FliO |
|  |  | *fliP* | 1773350-1774072+ | 722 | flagellar biosynthesis protein FliP |
|  |  | *fliQ* | 1774076-1774345+ | 269 | flagellar biosynthesis protein FliQ |
|  |  | *fliR* | 1774350-1775135+ | 785 | flagellar biosynthesis protein FliR |
|  |  | *flhB* | 1775139-1776275+ | 1136 | flagellar biosynthesis protein FlhB |
|  |  | *flhA* | 1776837-1778951+ | 2114 | flagellar biosynthesis protein FlhA |
|  |  | *flhF* | 1778963-1780279+ | 1316 | flagellar biosynthesis regulator FlhF |
|  |  | *motD* | 1787449-1788336+ | 887 | flagellar motor protein MotD |
|  |  | *flhB* | 1792861-1793190- | 329 | flagellar biosynthesis protein FlhB |
|  |  | *fliK* | 1793187-1794758- | 1571 | flagellar hook-length control protein FliK |
|  |  | *flgE* | 4442096-4443415- | 1319 | flagellar hook protein FlgE |
|  |  | *flgD* | 4443447-4444178- | 731 | flagellar basal body rod modification protein FlgD |
|  |  | *flgC* | 4444195-4444599- | 404 | flagellar basal body rod protein FlgC |
|  |  | *flgB* | 4444642-4445049- | 407 | flagellar basal body rod protein FlgB |
|  |  | *flgA* | 4448439-4449128+ | 689 | flagellar basal body P-ring biosynthesis protein FlgA |
|  |  | *flgM* | 4449270-4449587+ | 317 | flagellar biosynthesis anti-sigma factor FlgM |
|  |  | *flgN* | 4449636-4450103+ | 467 | flagellar biosynthesis protein FlgN |
|  | Fimbriae Related | *pilR* | 215285-216211+ | 926 | two-component response regulator PilR |
|  |  | *pilM* | 412156-412809+ | 653 | Type 4 fimbrial biogenesis protein PilM |
|  |  | *pilN* | 412809-413360+ | 551 | Type 4 fimbrial biogenesis protein PilN |
|  |  | *pilQ* | 414559-415890+ | 1331 | Type 4 fimbrial biogenesis protein PilQ |
|  |  | *pilF* | 1148403-1149161+ | 758 | Type 4 fimbrial biogenesis protein PilF |
|  |  | *fimA* | 4090182-4090742- | 560 | Type 1 fimbriae FimA |
|  |  | *pilC* | 5122953-5124179+ | 1226 | still frameshift type 4 fimbrial biogenesis protein PilC |
|  |  | *pilD* | 5124182-5125054+ | 872 | type 4 prepilin peptidase PilD |
|  |  | *FimU* | 5140018-5140596+ | 578 | Type 4 fimbrial biogenesis protein FimU |
|  |  | *pilJ* | 5612336-5614387- | 2051 | twitching motility protein PilJ |
|  |  | *pilI* | 5614384-5614920- | 536 | twitching motility protein PilI |
|  |  | *pilH* | 5614933-5615298- | 365 | twitching motility protein PilH |
|  |  | *pilG* | 5615343-5615750- | 407 | twitching motility protein PilG |
|  |  | *pilT* | 5623507-5624541- | 1034 | twitching motility protein PilT |
|  | Alginate Synthesis | *fleR* | 334979-336415- | 1436 | two-component response regulator FleR |
|  |  | *fleQ* | 3442670-3444076+ | 1406 | transcriptional regulator FleQ |
|  |  | *algD* | 4896803-4898119+ | 1316 | GDP-mannose 6-dehydrogenase AlgD |
|  |  | *algG* | 4898203-4899684+ | 1481 | alginate-c5-mannuronan-epimerase AlgG |
|  |  | *alg8* | 4899722-4900891+ | 1169 | alginate biosynthesis protein Alg8 |
|  |  | *alg44* | 4901065-4902435+ | 1370 | alginate biosynthesis protein Alg44 |
|  |  | *algK* | 4902432-4903919+ | 1487 | alginate biosynthetic protein AlgK |
|  |  | *algE* | 4903934-4905508+ | 1574 | outer membrane protein AlgE |
|  |  | *algX* | 4905532-4906968+ | 1436 | alginate biosynthesis protein AlgX |
|  |  | *algL* | 4906977-4908077+ | 1100 | poly(beta-d-mannuronate) lyase precursor AlgL |
|  |  | *algI* | 4908265-4909797+ | 1532 | alginate o-acetyltransferase AlgI |
|  |  | *algJ* | 4909809-4910999+ | 1190 | alginate o-acetyltransferase AlgJ |
|  |  | *algF* | 4911073-4911669+ | 596 | alginate o-acetyltransferase AlgF |
|  |  | *algA* | 4911817-4913268+ | 1451 | mannose-1-phosphate guanylyltransferase algA |
|  |  | *algQ* | 5788363-5788821- | 458 | alginate regulatory protein AlgQ |
|  |  | *algR* | 5794006-5794752- | 746 | alginate biosynthesis regulatory protein AlgR |
|  | c-di-GMP System | *deoR* | 745420-746688+ | 1268 | diguanylate cyclase (GGDEF domine) |
|  |  | *cpdA* | 1921176-1922933- | 1757 | c-di-GMP-specific phosphodiesterase class I (EAL domain), |
|  |  | *yahA* | 2793738-2795279- | 1541 | c-di-GMP-specific phosphodiesterase |
|  |  | *fabG* | 4348750-4349496+ | 746 | Cyclic-di-GMP-binding biofilm dispersal mediator protein |
|  |  | *ycgR* | 4450199-4450948+ | 749 | c-di-GMP-binding flagellar brake protein YcgR |
|  |  | *dncV* | 5465387-5466634- | 1247 | c-di-GMP synthetase |
| Spoilage Potential | Protease | *asP1* | 524375-524896+ | 521 | aspartyl protease Asp1 |
|  |  | *hflK* | 548251-549429+ | 1178 | membrane protease Hflk |
|  |  | *ftsH* | 853949-855859+ | 1910 | ATP-dependent metalloprotease FtsH |
|  |  | *degS* | 1023489-1024643- | 1154 | serine protease DegS |
|  |  | *aprX* | 1612570-1613802+ | 1232 | metalloprotease aprX |
|  |  | *aprA* | 1815635-1817236+ | 1601 | Alkaline protease AprA |
|  |  | *tsP* | 1923052-1925133- | 2081 | Tail-specific protease Tsp |
|  |  | *htpX* | 2091261-2092148- | 887 | protease HtpX |
|  |  | *prtC* | 2719879-2720874+ | 995 | Collagenase-like protease, PrtC |
|  |  | *serP* | 2983857-2986892+ | 3035 | serine protease serP |
|  |  | *clcP* | 3298103-3300682- | 2579 | ATP-dependent Clp protease clpP |
|  |  | *sprT* | 4424979-4425482+ | 503 | Zn-dependent metalloprotease sprT |
|  |  | *pmbA* | 5028609-5029955- | 1346 | Metalloprotease PmbA |
|  | Lipase | *estA* | 603442-604284+ | 842 | Triacylglycerol esterase estA |
|  |  | *rssA* | 1940727-1941881+ | 1154 | phospholipase RssA |
|  |  | *lipD* | 2979395-2980738+ | 1343 | Lipase D lipD |
|  |  | *apeE* | 5404682-5406511+ | 1829 | membrane lipase apeE |
|  |  | *pldB* | 5695004-5695945- | 941 | lysophospholipase pldB |
|  | Spoilage Related Metabolism | *cysP* | 205452-206462+ | 1010 | sulfate transport system substrate-binding protein CysP |
|  |  | *cysU* | 206630-207448+ | 818 | sulfate transport system permease protein cysU |
|  |  | *cysW* | 207462-208334+ | 872 | sulfate transport system permease protein cysW |
|  |  | *cysA* | 208338-209318+ | 980 | sulfate transport system ATP-binding protein cysA |
|  |  | *cysD* | 1025664-1026581+ | 917 | cysD sulfate adenylyltransferase |
|  |  | *cysN* | 1026591-1028489+ | 1898 | bifunctional enzyme CysN |
|  |  | *cysI* | 2811060-2812730+ | 1670 | sulfite reductase (NADPH) CysI |
|  |  | *cysK* | 4324885-4325799+ | 914 | cysteine synthase A cysK |
|  |  | *cysH* | 4343630-4344364+ | 734 | cysH phosphoadenosine phosphosulfate reductase |
|  |  | *cysJ* | 5100755-5103277+ | 2522 | cysJ sulfite reductase (NADPH) |
|  |  | *glpE* | 5430681-5431010+ | 329 | thiosulfate sulfurtransferase glpE |
|  |  | *sseA* | 5643694-5644548- | 854 | 3-mercaptopyruvate sulfurtransferase |
|  |  | *potI* | 5701051-5701935- | 884 | putrescine transport system permease protein PotI |
|  |  | *ssuD* | 5714741-5715889- | 1148 | ssuD alkanesulfonate monooxygenase |
|  |  | *aguB* | 285303-286181+ | 878 | N-carbamoylputrescine amidase |
|  |  | *thiI* | 339758-341155+ | 1397 | thiamine biosynthesis protein ThiI |
|  |  | *thiC* | 510966-512855+ | 1889 | thiamine biosynthesis protein ThiC |
|  |  | *ydeY* | 905385-906398+ | 1013 | Periplasmic DMSO reductase |
|  |  | *ydeZ* | 906398-907018+ | 620 | Periplasmic TMAO reductase |
|  |  | *iscS* | 1141640-1142854+ | 1214 | cysteine desulfurase iscS |
|  |  | *speE* | 1618686-1619372- | 686 | spermidine synthase |
|  |  | *ordL* | 3890782-3892080+ | 1298 | gamma-glutamylputrescine oxidase |
|  |  | *potF* | 4076717-4077802- | 1085 | putrescine-binding periplasmic protein E |
|  |  | *argD* | 4477489-4478709- | 1220 | N-succinyldiaminopimelate aminotransferase |
|  |  | *arcA* | 4578130-4579386+ | 1256 | Arginine deiminase E |
|  |  | *thiO* | 5136532-5137632- | 1100 | thiO glycine oxidase |
|  |  | *thiD* | 5228985-5229782+ | 797 | thiD phosphomethylpyrimidine kinase |
|  |  | *thiE* | 5229801-5230424+ | 623 | thiE thiamine-phosphate pyrophosphorylase |
|  |  | *hemL* | 5230442-5231725+ | 1283 | hemL glutamate-1-semialdehyde 2,1-aminomutase |
|  |  | *tmD* | 5235897-5237690- | 1793 | trimethylamine dehydrogenase |
|  |  | *thiL* | 5318106-5319071- | 965 | thiamine-monophosphate kinase |
|  |  | *thiG* | 5632123-5632917- | 794 | thiamine biosynthesis ThiG |
|  |  | *spuC* | 5706686-5708086- | 1400 | putrescine aminotransferase |
| Stress Response | Environmental Stress Response | *cusS* | 937305-938690- | 1385 | heavy metal sensor histidine kinase CusS |
|  |  | *rpoS* | 1376611-1377615+ | 1004 | polymerase sigma factor RpoS |
|  |  | *relA* | 1692553-1694796+ | 2243 | stringent stress response RelA |
|  |  | *bsaA* | 2094410-2094895+ | 485 | Glutathione peroxidase |
|  |  | *pvdE* | 2290240-2291889- | 1649 | Pyoverdine biosynthesis protein |
|  |  | *pvdA* | 2302471-2303808+ | 1337 | L-ornithine N5-oxygenase |
|  |  | *ahpC* | 4753995-4754597- | 602 | Alkyl hydroperoxide reductase subunit AhpC |
|  |  | *czcR* | 4937598-4938275+ | 677 | Heavy metal response regulator |
|  |  | *sspB* | 5056434-5056847- | 413 | Stringent starvation protein B |
|  |  | *sspA* | 5056865-5057482^-^ | 617 | Stringent starvation protein A |
|  |  | *merR* | 5306256-5306654^+^ | 398 | MerR family transcriptional regulator |

Fig. S1


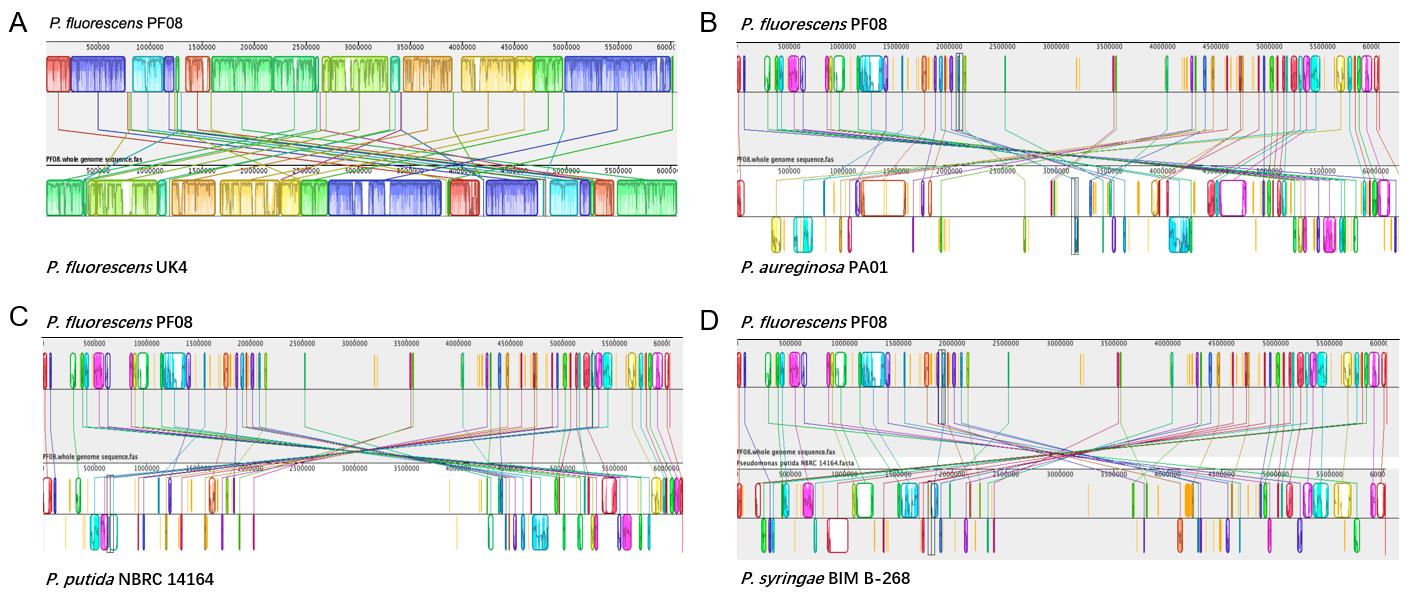


Fig. S2


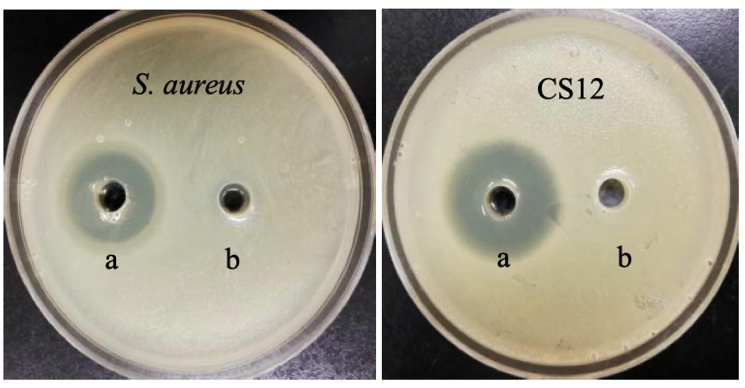


**References**

Berlin, K., Koren, S., Chin, C.S., Drake, J.P., Landolin, J.M., Phillippy, A.M. 2015. Assembling large genomes with single-molecule sequencing and locality-sensitive hashing, Nat. Biotechnol. 33(6), 623–630 [doi:[10.1038/nbt.3238](https://doi.org/10.1038/nbt.3238)].

Koren, S., Phillippy, A.M. 2015, One chromosome, one contig: complete microbial genomes from long-read sequencing and assembly, Curr. Opin. Microbiol. 23, 110–120 [doi:[10.1016/j.mib.2014.11.014](https://doi.org/10.1016/j.mib.2014.11.014)].

Li, T., Wang, D., Ren, L., Mei, Y., Ding, T., Li, Q., Chen, H., Li, J. 2019. Involvement of exogenous N-acyl-homoserine lactones in spoilage potential of *Pseudomonas fluorescens* isolated from refrigerated turbot, Front. Microbiol. 10, 2716 [doi:10.3389/fmicb.2019.02716].
